# OCT Amplitude and Speckle Statistics of Discrete Random Media

**DOI:** 10.1038/s41598-017-14115-3

**Published:** 2017-11-01

**Authors:** Mitra Almasian, Ton G. van Leeuwen, Dirk J. Faber

**Affiliations:** Academic Medical Center, University of Amsterdam, Department of Biomedical Engineering and Physics, Amsterdam, The Netherlands

## Abstract

Speckle, amplitude fluctuations in optical coherence tomography (OCT) images, contains information on sub-resolution structural properties of the imaged sample. Speckle statistics could therefore be utilized in the characterization of biological tissues. However, a rigorous theoretical framework relating OCT speckle statistics to structural tissue properties has yet to be developed. As a first step, we present a theoretical description of OCT speckle, relating the OCT amplitude variance to size and organization for samples of discrete random media (DRM). Starting the calculations from the size and organization of the scattering particles, we analytically find expressions for the OCT amplitude mean, amplitude variance, the backscattering coefficient and the scattering coefficient. We assume fully developed speckle and verify the validity of this assumption by experiments on controlled samples of silica microspheres suspended in water. We show that the OCT amplitude variance is sensitive to sub-resolution changes in size and organization of the scattering particles. Experimentally determined and theoretically calculated optical properties are compared and in good agreement.

## Introduction

Changes in the structural properties of tissue, such as the size and organization of cells and cellular components, are important indicators of disease development and progression. As these changes will alter the interaction of light with the tissue, their quantification employing optical techniques could enable minimally-invasive detection, early-stage diagnosis, and monitoring of disease progression and treatment. Therefore, clinical studies aim to quantify tissue optical properties for the purpose of tissue characterization^1–6^. Yet, the relation between structural properties and optical properties is less understood. Optical coherence tomography (OCT) is a technique which allows for measurement of tissue optical properties in a spatially confined region, corresponding to the OCT resolution; OCT-signal parameters amplitude and attenuation are determined as a measure for sample optical properties. In our previous work we proposed and validated a reliable approach to obtain the amplitude and attenuation coefficient from OCT data. By carefully taking into account system parameters such as confocal point spread function and sensitivity roll-off, and the effects multiple scattering, we arrived at values for sample optical properties: backscattering coefficient (*µ*_*b,NA*_) and scattering coefficient (*µ*_*s*_)^7^. In addition to the amplitude and the attenuation coefficient, studies suggest that statistical measures of the speckle pattern in OCT images can be quantified as additional parameters to derive sub-resolution structural tissue properties, and potentially provide for a tool for tissue characterization in the clinic^8,9^. Speckle in OCT is the voxel-to-voxel fluctuation of the OCT amplitude caused by phase differences of the backscattered field due to the spatial distribution of the scattering particles in the sample^10^. Hence, speckle contains information on the scattering particles that are in general smaller than the OCT resolution of approximately 5–20 µm. Speckle-based flow measurements (e.g. in flow channels or blood vessels) have been the focus of a large number of studies, in which flow information is usually obtained by quantifying the speckle de- or auto-correlation between successive scans of a fixed tissue location^11,12^. Here, we will focus on the characterization of static speckle as a measure for structural tissue properties. Speckle, which from now on we will refer to as the OCT amplitude distribution, can be studied by analyzing the distribution of the amplitude values of a region of interest (ROI) in the OCT image. From the amplitude distribution, values for the mean <A>, the standard deviation (σ), the variance (σ^2^) and the contrast (c = σ/<A>) can be calculated. The amplitude distribution can also be fitted with a probability distribution function, from which the function shape parameters can be obtained. As a first step towards modelling the amplitude distribution from actual tissue, we start with an analysis of discrete random media (DRM). For samples with randomly positioned identical scattering particles within the sampling, or coherence volume V_C_ defined below, the backscattered field phases are uniformly and randomly distributed between 0 and 2π^13^. For approximately >10 scattering particle the amplitude distribution for the linear representation of the OCT signal is assumed to follow a Rayleigh distribution^14^. For less than approximately 10 particles in V_C_ contributing, it has been shown that the contrast of the OCT amplitude distribution is proportional to particle concentration^14,15^. In this study, we focus on samples with a density of more than 10 scattering particles in the coherence volume V_C_. While different models have been proposed to describe the OCT amplitude distribution in order to characterize tissue^8,11,15–17^, a rigorous theoretical framework relating the OCT amplitude distribution to structural tissue properties, such as size and organization, has yet to be developed.

In this paper we therefore aim to study the relationship between the OCT amplitude, its variance and the sample optical and structural properties. Starting from the properties of the scattering particles, e.g. size, concentration and refractive index, we derive expressions for the OCT signal, the backscattering coefficient within the detection NA (*µ*_*b,NA*_) and scattering coefficient (*µ*_*s*_), for discrete random media (DRM). Assuming the OCT amplitude to be Rayleigh distributed, we find expressions for the amplitude mean and amplitude variance in terms of sample optical properties. These expressions are then used to study the relation between retrieved optical properties and particle size and concentration. Experimentally, we study the dependence of the OCT amplitude and it’s variance on sub-resolution changes in size and concentration of the scattering particles, by probing controlled samples of silica microspheres suspended in water with equal *µ*_*s*_, but different scattering particle size. From the OCT data of these samples we determine the attenuation coefficient and amplitude variance. After correcting for system parameters we compare the experimentally determined and the theoretically predicted values of the optical properties. Furthermore, we use the OCT data to validate the assumption of Rayleigh distributed OCT amplitudes and to study the influence of the ROI window selection on the amplitude distribution.

### Derivation of the OCT signal of DRM

We derive the OCT signal for discrete random media (DRM) containing mono-sized spherical scattering particles that are randomly distributed throughout the sample. First, we determine the organization of the scattering particles within the OCT coherence volume (*V*_*C*_), defined as the volume of an ellipsoid in equation (1)^15^.

1 $${V}_{C}=\frac{4\pi {\omega }_{0}^{2}\sqrt{2\,{\rm{ln}}(2)}{L}_{C}}{6}$$

where *ω*_0_ is the 1/e waist of the beam illuminating the sample and *L*_*c*_ is the coherence length measured as the FWHM of the axial width of the coherence function in the medium. In our experiments, *V*_*C*_ of ~20 × 10^3^ μm^3^ with the average number of particles in the *V*_*C*_ for our system per sample is given in the Methods section. It is assumed that for a given location in the sample, only pa\rticles from within the coherence volume around that location contribute to the OCT signal.

**Table 1:** Overview of the samples: mean and standard deviation of the diameter of the silica microspheres determined using transmission electron microscopy (TEM), volume fraction, and the estimated number of silica microspheres in the scattering voxel.

sample	mean diameter (µm) TEM	Volume Fraction (f_v_)	# microspheres in Vc	*μ*_s_ (mm^−1^)	amplitude variance (a.u.)	*μ*_b,NA_ (mm^−1^)
A	0.47 ± 0.03	0.11 ± 0.006	~4.0*10^4^	3.8 ± 0.11	1197 ± 37	9.5 ± 0.28 *10^−5^
B, F	0.70 ± 0.03	0.04 ± 0.002	~4.7*10^3^	4.5 ± 0.08	148 ± 9	1.2 ± 0.07 *10^−5^
		0.08 ± 0.004	~9.1*10^3^	6.38 ± 0.24	262 ± 12	2.1 ± 0.06 *10^−5^
C, G	0.91 ± 0.02	0.03 ± 0.002	~1.6*10^3^	4.24 ± 0.13	267 ± 7	2.1 ± 0.005 *10^−5^
		0.06 ± 0.003	~3.1*10^3^	6.39 ± 0.22	503 ± 15	4.0 ± 0.1 *10^−5^ (calibration)
D, H	1.60 ± 0.03	0.02 ± 0.0001	~1.9*10^2^	4.26 ± 0.20	12 ± 1	9.5 ± 0.9 *10^−7^
		0.04 ± 0.002	~3.9*10^2^	6.11 ± 0.30	38 ± 2	3.0 ± 0.2 *10^−6^

Experimentally obtained scattering coefficient (*μ*_S_) expressed as the mean and standard deviation obtained from five B-scans and the experimentally obtained amplitude variance, and backscattering coefficient expressed as the mean and standard deviation obtained from ten B-scans per sample.

**Figure 1:**
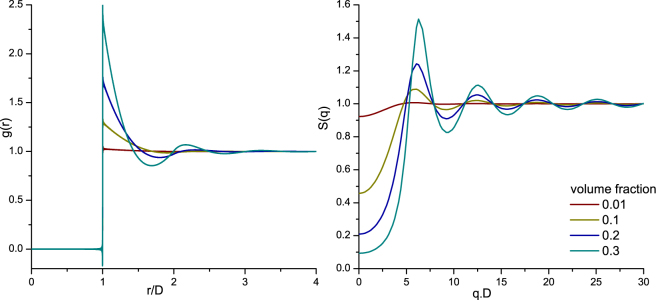
Pair correlation function (left) of mono-disperse DRM for increasing volume fractions (0.01, 0.1, 0.2 and 0.3). On the horizontal axis, the distance between particles (r) is normalized on particle diameter (D). The corresponding structure factor (right), where q is the scattering vector.

The spatial distribution of the particles can be described by the average density ρ and pair correlation function $g(\mathop{r}\limits^{\rightharpoonup },{\mathop{r}\limits^{\rightharpoonup }}^{^{\prime} })$ ^18^.

2 $$g(\mathop{r}\limits^{\rightharpoonup },{\mathop{r}\limits^{\rightharpoonup }}^{^{\prime} })=\frac{\langle {\sum }_{i=1}^{N}{\sum }_{j\ne i}^{N}\delta (\mathop{r}\limits^{\rightharpoonup }-{\mathop{r}\limits^{\rightharpoonup }}_{i})\delta ({\mathop{r}\limits^{\rightharpoonup }}^{^{\prime} }-{\mathop{r}\limits^{\rightharpoonup }}_{j})\rangle }{{\rho }^{2}}$$

where ρ is the average particle density, N is the number of particles and $\delta (\mathop{r}\limits^{\rightharpoonup }-{\mathop{r}\limits^{\rightharpoonup }}_{i})$ is a delta function giving the position of the i’th particle. When the medium is homogeneous and isotropic, the pair correlation function depends only on the distances between particles e.g. $| {\rm{\Delta }}\mathop{r}\limits^{\rightharpoonup }| =| {\mathop{r}\limits^{\rightharpoonup }}_{2}-{\mathop{r}\limits^{\rightharpoonup }}_{1}|$ and an analytical solution can be found. In this case the pair correlation function can be interpreted as a probability distribution for distances between particles. It is normalized on the probability distribution of particle separations without any constraints to particle placement, so that g(r) → 1 for large particle separations. The Percus-Yevick solution^19^ to the pair correlation function for three volume fractions (*f*_v_ = ρ.V_particle_) is depicted in the left panel of Fig. 1, in which the horizontal axis is the distance between particles normalized on particle diameter. Note the ‘forbidden’ region of particle separations of r/D < 1 for all volume factions because hard spherical particles cannot overlap in space. With increasing volume fraction the probability of finding a 2^nd^ particle near a 1^st^ particle increases, leading to increasing g(r) above at multiples of the diameter and decreasing g(r) below unity in between. Placing particles at higher multiples of the diameter is easier than placing at 1x or 2x the diameter. Therefore, the amplitude of the peaks decrease and the peaks become broader at increasing value of r. This organization of scattering particles in the sample is reflected in the light scattering pattern via the Structure factor S(q), where $q=\frac{4\pi }{\lambda }\,sin\,\frac{1}{2}\theta$; with λ the wavelength and θ the scattering angle (Fig. 1, right panel). The structure factor forms a Fourier-pair with the pair correlation function, and serves as a volume-fraction dependent weighting factor on the angular scattering pattern of the individual particles in the sample. Note that the horizontal axis is multiplied with diameter for scaling. For extremely dilute samples S(q) equal unity over the whole domain, indicating that the angular scattering pattern from the sample is identical to that of the individual particles.

Second, we describe the complex OCT interference signal in the spatial domain, which is given by *x*(*z*) + *i y*(*z*). The geometry and OCT signal description for this derivation are given in Supplementary Section I. Here, *x*(*z*) is detected directly in time domain OCT or is obtained from the real part of the Fourier transform of the spectral interferogram in spectral domain OCT (thus, the imaginary part of the complex OCT signal *y*(*z*) can be obtained from *x*(*z*) through Hilbert transform in time-domain OCT or as the imaginary part of the Fourier transform in spectral domain OCT, see Supplementary Section II).

Optical path length z is measured from ‘zero delay’, i.e. the position at which optical path lengths in sample arm and reference arm are equal. The real part x(z) and imaginary part y(z) are cosine (resp. sine) modulated signals with zero mean, and equal, non-zero variance. The OCT amplitude ‘A-line’ is found from the envelope of *x*(*z*) and *y*(*z*) by: A(z) = √(*x* ^2^(*z*) + *y* ^2^(*z*)). Commonly, the logarithmic value of A(z) is plotted to obtain OCT images. The random positions of the particles in the coherence volume (*V*_*C*_) implies that for N > 10 the field collected from the sample arm follows the statistics of a random phasor sum and that the OCT amplitude is Rayleigh distributed^13^ (Supplementary Section III). This amplitude distribution *p(A*) is described in (equation(3)). The mean <A> and the variance ${\sigma }_{A}^{2}$ of the OCT amplitude are given by equation (4), where σ^2^_x,y_ denotes the variance of x(z) and y(z), respectively. This yields that the contrast of the amplitude, the ratio of standard deviation over mean, in OCT is $\sqrt{\frac{4}{\pi }-1}\approx 0.52$ ^14,20^.

3 $$p(A)=\frac{A}{{\sigma }^{2}}{e}^{-{A}^{2}/(2{\sigma }^{2})}$$

4 $$\langle A\rangle =\sqrt{\frac{\pi }{2}{\sigma }_{x,y}^{2}};\,{\sigma }_{A}^{2}=(2-\frac{\pi }{2}){\sigma }_{x,y}^{2}$$

We will now pursue an expression for the OCT amplitude mean and variance in terms of the optical properties of the DRM. This requires calculation of x(z) and its variance. To simplify calculations, we assume the reference mirror is perfectly matched with one reference particle in the sample (the position of this reference particle in depth direction is set equal to the optical path length of the reference arm, Supplementary Fig. SI 1). All subsequent phase differences induced by positions within the coherence volume (*V*_*c*_) are calculated with respect to this reference particle (see Supplementary Section I). We further assume that, by the 1^st^ Born approximation, all particles within V_C_ experience the same incident field.

To arrive at an expression for the real part of the OCT signal, x(z), the complex coherence function γ(z) (the Fourier transform of the light source spectrum) is convolved with the reflectivity profile of the sample (a simulated example is shown in Supplementary Fig. SI 2). The latter term may be described in terms of δ-functions of particle position^21^. The contribution to *x(z)* from each individual particle is found by evaluating the scattered field over the collection solid angle ΩNA determined by the numerical aperture (NA) of the OCT system. In terms of the scattering vecto $\mathop{q}\limits^{\rightharpoonup }=\mathop{q}\limits^{\rightharpoonup }(\theta )$ r, for which the magnitude is given by $| \mathop{q}\limits^{\rightharpoonup }| =2k\,sin(\theta /2)$ where *θ* is the scattering angle, the expression for x(z) becomes:

5 $$x(z)\propto {\rm{Re}}\{{\int }_{\Omega NA}\sum _{i}\gamma (\mathop{q}\limits^{\rightharpoonup }\cdot {\mathop{r}\limits^{\rightharpoonup }}_{i}){e}^{i\mathop{q}\limits^{\rightharpoonup }\cdot {\mathop{r}\limits^{\rightharpoonup }}_{i}\frac{f(\theta ,\phi )}{kR}}d\Omega \}$$

where, *f(θ,ϕ)* is the field scattering amplitude which can be calculated using Mie theory for spherical particles^22^, *R* is the distance from particle to detection lens (which we assume approximately equal for all particles), ${\mathop{r}\limits^{\rightharpoonup }}_{i}$ is the position of particle *i* with respect to the reference particle. The exponential term describes the phase difference $\mathop{q}\limits^{\rightharpoonup }\cdot {\mathop{r}\limits^{\rightharpoonup }}_{i}$ between particle *i* and the reference particle. Since only particles within the coherence volume (*V*_*c*_) contribute to the signal, we further simplify the coherence gating term in this equation by setting γ = 1 and sum over *N* = *ρV*_*C*_ particles, where *ρ* is the density of particles. For discrete random media, the mean of Eq. 5 <x(z)> is 0. A detailed derivation leading to equation (5) is given in Supplementary Section IV.

To calculate the mean OCT amplitude <A>, equation (4), we proceed to calculate the variance of *x(z*) from equation (5). A detailed derivation leading to equation (6) is given in Supplementary Section V. In equation (6) we have expressed the integration over the solid angle of the detection NA in spherical coordinates, and used the fact that all particles are identical, so that we can make the following substitution for the squared scattering amplitude in terms of the differential scattering cross section ${\sigma }_{scat}(\theta ,\phi )=f{(\theta ,\phi )}^{2}/{k}^{2}$:

6 $${\sigma }_{x}^{2}=\langle x{(z)}^{2}\rangle \propto {\rm{Re}}\{{\int }_{0}^{2\pi }{\int }_{(\pi -NA)}^{\pi }\langle {\sum }_{i=1}^{N}{\sum }_{j=1}^{N}{e}^{i\mathop{q}\limits^{\rightharpoonup }\cdot {\mathop{r}\limits^{\rightharpoonup }}_{i}}{e}^{-i\mathop{q}\limits^{\rightharpoonup }\cdot {\mathop{r}\limits^{\rightharpoonup }}_{j}}\rangle {\sigma }_{scat}(\theta ,\phi )sin\,\theta d\theta d\phi \}$$

Note that for spherical particles at the sizes considered here, the differential scattering cross section does not depend on φ so that integration over φ yields a factor 2π only. Following Hansen and MacDonald^18^, the double sum between chevrons 〈…〉 can be expanded as:

7 $$N\frac{1}{N}\langle {\sum }_{i=1}^{N}{\sum }_{j=1}^{N}{e}^{i\mathop{q}\limits^{\rightharpoonup }\cdot {\mathop{r}\limits^{\rightharpoonup }}_{i}}{e}^{-i\mathop{q}\limits^{\rightharpoonup }\cdot {\mathop{r}\limits^{\rightharpoonup }}_{j}}\rangle =\rho {V}_{C}S(q)=\rho {V}_{C}(1+\rho \int g({\rm{\Delta }}r)ei\mathop{q}\limits^{\rightharpoonup }\cdot \Delta \mathop{r}\limits^{\rightharpoonup }d{\rm{\Delta }}\mathop{r}\limits^{\rightharpoonup })$$

where *S(q*), or more conveniently *S(θ)* is the structure factor obtained as the Fourier transform of the pair correlation function *g(Δr)*. With these substitutions, equation 6 then simplifies to:

8 $${\sigma }_{x}^{2}=\propto \rho {V}_{C}\times 2\pi {\int }_{(\pi -NA)}^{\pi }{\sigma }_{scat}(\theta )S(\theta )sin\,\theta d\theta $$

Equation (8) shows that the variance of x(z) and, by equation (4), the mean and variance of the OCT amplitude contain information of the individual scatterer (through the differential scattering cross section), their density ρ and organization (through the structure factor). Comparing the integral in Equation (8) with the definition of the total scattering cross section σ_s_ (integral of the differential scattering cross section over the full angular domain) and the definition of the scattering coefficient μ_s_ = ρσ_s_; suggests the definition of the “backscatter coefficient within the NA” as follows:

9 $${\mu }_{b,NA}=\rho 2\pi {\int }_{(\pi -NA)}^{\pi }{\sigma }_{scat}(\theta )S(\theta )sin\,\theta d\theta $$

So that ${\sigma }_{x}^{2}\propto {\mu }_{b,NA}$ and ${\sigma }_{A}^{2}\propto {\mu }_{b,NA}$ (Supplementary Section VI). Theoretically, the NA could be increased to capture all scattered light. Analogous to the derivation leading to equation (9), this gives the total scattering coefficient:

10 $${\mu }_{s}=\rho 2\pi {\int }_{0}^{\pi }{\sigma }_{scat}(\theta )S(\theta )sin\,\theta d\theta $$

where the only difference between the ‘conventional’ expression for the scattering coefficient is the inclusion of the structure factor, weighting the angular distribution of scattered light to account for particle organization in the sample. The proportionality in Equations (5), (6), and (8) depends on a number of system properties: the power in the sample and reference arms, the detector quantum efficiency, and z-dependent factors such as the confocal point spread function and sensitivity roll-off for spectral domain OCT systems. We assume that these can be determined by calibration measurements and therefore introduce the factor α(z)^7^. The proportionality also depends on signal losses due to light scattering towards and from the backscatter position, which is accounted for by the scattering coefficient in equation (10). (see Supplementary Section VI for more detailed discussion).

By combing equation (4) and equation (8), we proceed to write the mean and the variance of the OCT signal amplitude as a function of sample properties:

11 $$\langle A(z)\rangle =\sqrt{\alpha (z)\frac{\pi }{2}{\mu }_{b,NA}{V}_{C}\,exp(-2{\mu }_{s}(z-{z}_{0}))}$$

12 $${\sigma }_{A}^{2}(z)=\alpha (z)(2-\frac{\pi }{2}){\mu }_{b,NA}{V}_{C}\,exp(-2{\mu }_{s}(z-{z}_{0}))$$

where z_0_ is the axial distance from zero-delay to the sample boundary. The exponential factor accounts for light attenuation to and from the reference particle, where z is now measured from zero delay.

We have thus derived the OCT amplitude (equation (11)) and amplitude variance (equation (12)) as functions of depth and the optical properties *µ*_*s*_ and *µ*_*b,NA*_, which are in turn expressed in terms of the scattering cross section (*σ*_*scat*_) and structure factor (*S(θ)*) in equations (9) and (10). Through the initial assumption of a homogeneous, isotropic medium, both *µ*_*b,NA*_ and *µ*_*s*_ have no z-dependence in the sample.

## Results

### Optical properties of DRM

**Figure 2:**
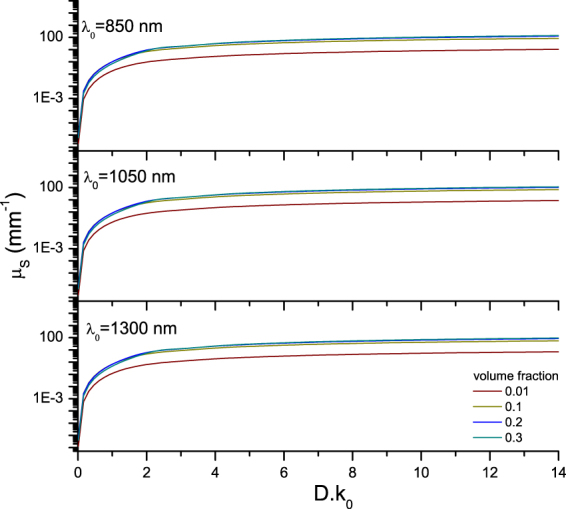
Calculated scattering coefficient (*µ*_*s*_, equation (10)) as a function of optical particle diameter (D. k_0_, k_0_ = 2π/λ_0, vacuum_) for increasing volume fractions (0.01, 0.1, 0.2 and 0.3) at three common OCT wavelengths and bandwidths: λ_0_ = 850 (∆λ 65) nm, λ_0_ = 1050 (∆λ 81) nm and λ_0_ = 1300 (∆λ 100) nm. The used refractive index of the medium and silica beads were 1.324 and 1.425 at 1300 nm, respectively. The refractive indices were varied with wavelength and bandwidth for the calculations. The magnitude of *µ*_*s*_ decreases with increasing wavelength, and increases non-linearly with an increase in scattering particle concentration.

**Figure 3:**
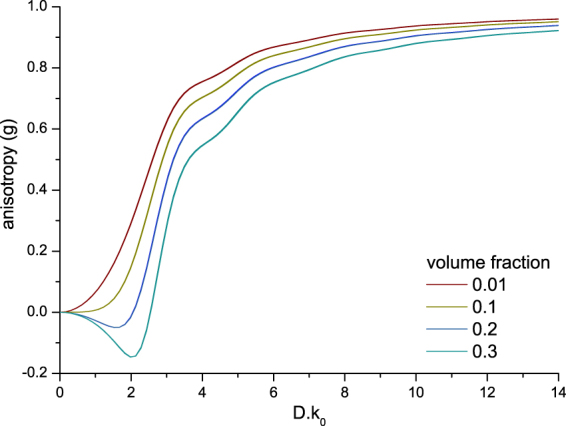
Calculated scattering anisotropy (g) as a function of optical particle diameter (D. k_0_, k_0_ = 2π/λ_0_, vaccuum) for increasing volume fractions (0.01, 0.1, 0.2 and 0.3) at three common OCT wavelengths and bandwidths: λ_0_ = 850 (∆λ 65) nm, λ_0_ = 1050 (∆λ 81) nm and λ_0_ = 1300 (∆λ 100) nm (the curves of the different wavelengths overlap). The used refractive index of the medium and silica beads were 1.324 and 1.425 at 1300 nm respectively, the refractive indices were varied with wavelength and bandwidth for the calculations. The anisotropy (g) is calculated as the average cosine of the concentration-dependent phase function. Note that for high volume fractions, g can become negative indicating a predominantly backward angular scattering pattern.

**Figure 4:**
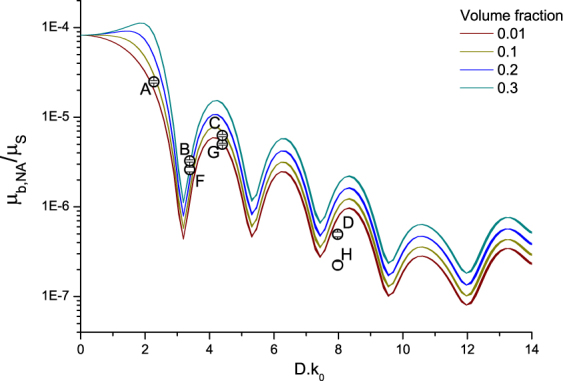
Calculated ratio of the backscattering coefficient over the scattering coefficient (µ_b,NA_ / µ_s_, equations (9) and (10)) as a function of optical particle diameter (D.k0, k_0_ = 2π /λ_0_,vacuum) for increasing volume fractions (0.01, 0.1, 0.2 and 0.3) at three common OCT wavelengths and bandwidths: λ_0_ = 850 (∆λ 65) nm, λ_0_ = 1050 (∆λ 81) nm and λ_0_ = 1300 (∆λ 100) nm (the curves of the different wavelengths overlap). The used refractive index of the medium and silica beads were 1.324 and 1.425 at 1300 nm respectively, the refractive indices were varied with wavelength and bandwidth for the calculations. The typical Mie oscillatory pattern in the backscatter coefficient is clearly present. The black circles indicate the experimental data points A-H and the corresponding error bars.

Equations (9) and (10) were used to calculate the values of the scattering coefficient (*µ*_*s*_) and the backscattering coefficient (*µ*_*b,NA*_) as a function of particle size and concentration. For completeness, the scattering anisotropy (g) is also calculated. The results are plotted as a function of optical size (*D.k*_0_ *, k*_0_ = 2*π*/*λ*_0*,vaccuum*_) for four volume fractions of scattering particles (0.01, 0.1, 0.2 and 0.3) at three commonly used OCT wavelengths with a Gaussian bandwidth in Figs 2, 3 and 4. The center wavelengths (*λ*_0_) and FWHM bandwidths ∆*λ*, which is a fixed ratio of the central wavelength, used for the calculations are: 850 (∆*λ* 65) nm, 1050 (∆*λ* 81) nm and 1300 (∆*λ* 100) nm. The *µ*_*s*_ curves are plotted in Fig. 2. The magnitude of *µ*_*s*_ decreases with increasing wavelength, and increases non-linearly with an increase in scattering particle concentration. The scattering anisotropy (*g*), which is a measure for the directionality of the phase function and is defined by < cos *(θ)* > of the phase function, is plotted in Fig. 3. Note, that contrary to Mie-predictions (Supplementary Section VII) the anisotropy changes with volume fraction. This concentration-dependent effect is taken into account by the addition of the structure factor to the Mie-based calculation of the phase function. Contrary to linear upscaling of the Mie-predictions, when the concentration-dependent effects are taken into account, the shape of the phase function changes with concentration (Supplementary Section VIII). For the lowest volume fraction of 0.01, the structure factor *S(θ)* (right panel Fig. 1) converges to unity and the integral part of equations (9) and (10) converge to the concentration independent expressions. The ratio of *µ*_*b,NA*_ over *µ*_*s*_, which describes the fraction of backscattered photons within the system NA independent of scattering efficiency and density of the scattering particles, also called *P*_*NA*_, is plotted Fig. 4. The periodic Mie oscillations are clearly visible in Fig. 4.

The OCT attenuation coefficient and amplitude variance of the silica microsphere samples were obtained from the OCT data. After calibration for system parameters (roll-off and point spread function) the attenuation coefficient is assumed equal to *µ*_*s*_^7^. Here, we correct for water absorption at 1300 nm, and assume minimal contribution of multiple scattering in the studied samples. The results of the experimentally determined values of *µ*_*s*_ and amplitude variance are summarized in Table 1. We found that samples with (approximately) equal *µ*_*s*_, but differently sized silica microspheres had different values for the amplitude variance. To facilitate comparison between the experimental and calculated optical properties, the experimental values of the amplitude variance are scaled to the calculated *µ*_*b*,*NA*_ values using a scaling factor derived from data point G. The experimentally determined ratio of *µ*_*b,NA*_ and *µ*_*s*_ for samples A to H are plotted in Fig. 4 together with the calculated curves of *µ*_*b,NA/*_ *µ*_*s*_.

### OCT amplitude distribution

**Figure 5:**
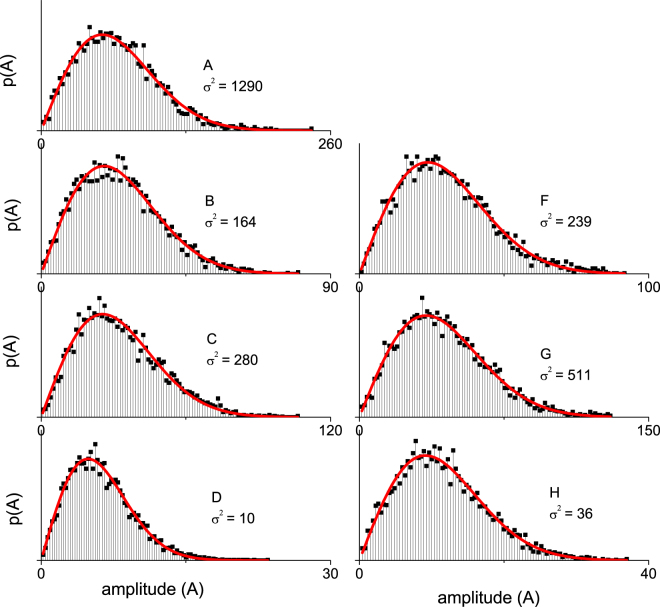
Amplitude histograms of the samples within the region of interest fitted with a Rayleigh distribution (Eq. 3). The R^2^ values for all fits are between 0.98 and 0.99. The R^2^ and amplitude variance (σ^2^) from the fit are given per sample. The Region of interest was 0.08 mm in depth and ~3.5 mm in width, starting at 0.03 mm optical path length below the cuvette-sample interface.

The amplitude distributions p(A) obtained from the same ROI window which also yielded the values for the amplitude variance of samples A–H (Table 1), are plotted in Fig. 5. The amplitude histograms are normalized such that the area under the curve equals 1, the histogram is then fitted with a Rayleigh distribution (equation (3)). The corresponding goodness of the fit R^2^ and the variance (σ^2^) of the fit are reported.

### Influence of the ROI window on the OCT amplitude distribution

**Figure 6:**
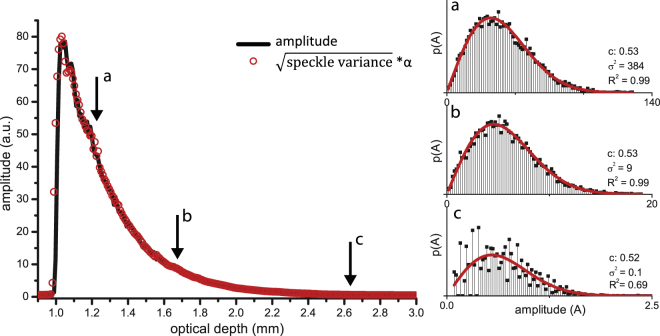
Left panel: square root of the amplitude variance from within a ROI window of 0.08 mm in depth, ~3mm in width, moved in depth (red circles) and mean amplitude of the OCT signal (black line) of sample A. A single scaling factor (𝛼) is used to scale the square root of the amplitude variance to the mean amplitude. Panels on the right side shows the histograms of the amplitude distributions within the ROI from points a-c as indicated with arrows in the left panel. The amplitude distributions are fitted with a Rayleigh distribution (equation (3)), giving the reported contrast, speckle variance and R^2^ of the fits. The amplitude stays Rayleigh distributed when the ROI is moved in depth, until regions of very low SNR are reached (point c).

The influence of the ROI window on the amplitude distribution, variance and contrast as well as the relation between amplitude variance and the mean amplitude of the OCT signal (equation (4)) were studied by varying the location and size of the ROI windows. Figure 6 summarizes the results for a ROI window (0.08 mm in depth) which was moved down along the depth-axis in the OCT image. The lateral dimensions of the ROI were chosen such that the amplitude contrast equaled 0.52. The square root of the amplitude variance is proportional to the amplitude of the OCT image as described by equation (4).

**Figure 7:**
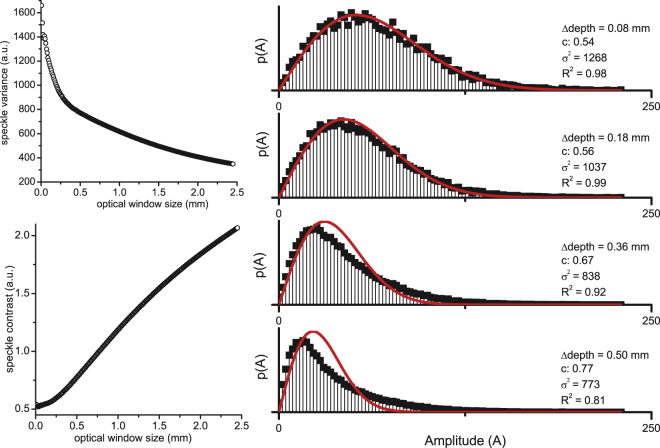
Change in amplitude contrast vs. ROI size along the depth axis, of sample A. Left panels show the amplitude variance (**a**) and contrast (**b**) for a ROI window that is enlarged in size in depth, starting at 0.03 mm optical path length below the cuvette-sample interface. ROI width was ~3.5 mm. In the right panel: amplitude histograms of four specific window sizes (0.08 mm, 0.18 mm, 0.36 mm, 0.50 mm optical path length).

Figure 7 summarizes the results for a ROI window which was increased in size along the depth-axis. The amplitude variance decreases as the window size increases. Conversely, the amplitude contrast increases with increasing window size and deviates from the theoretical value of 0.52: the contrast value exceeds 0.6 for ROI windows larger then 1/*µ*_*s*_ in depth. The amplitude distributions corresponding to four ROI window sizes are plotted and show that the distribution deviates from a Rayleigh as strongly attenuated (lower amplitude) signal contributes to the amplitude distribution from within the ROI window.

## Discussion

In this paper we present an analytical derivation of the OCT signal of discrete random media as a function of structural and optical sample properties. To this end, we express the OCT signal in depth as a function of sample optical properties (*µ*_*s*_ and *µ*_*b,NA*_) which are in turn calculated from the following sample properties: particle size, concentration and refractive index. Further, the effect of nonlinear scaling of optical properties with volume fraction (concentration-dependent scattering^7,23^) as expressed by the structure factor S(θ) follows directly from the consideration of both intensity and phase of the backscattered sample field. Figures 2, 3 and 4 show the concentration-dependent *µ*_*s*_ *, µ*_*b,NA*_ *, µ*_*b,NA*_ */µ*_*s*_ ratio and scattering anisotropy calculated with equations (9) and (10) as a function of particle size at different volume fractions. Consistent with our previous work^7,23^ we see that the concentration-dependent results (caused by inclusion of the structure factor) differ from the Mie-predictions (Supplementary Section VII): the scattering coefficient and backscatter coefficient increase, with increasingly slower rate with concentration. Second, the structure factor causes an angular redistribution of the scattered light (Supplementary Section VIII), effectively causing the backscatter coefficient to increase at a faster rate than the total scattering coefficient.

To arrive at an expression for the OCT signal amplitude and its variance (i.e. speckle) we assume the OCT amplitude to be Rayleigh distributed^13,14,16,20^. While in literature the OCT amplitude distribution has been modelled using various distribution functions^8,17^, a rigorous physical framework for the OCT amplitude distribution has yet to be developed. Krillin *et al*.^17^ performed Monte Carlo simulations in which the phase of the photons is tracked, and compare the simulated speckle with experimental OCT data of polystyrene microspheres suspended in water. They show that the experimental and simulated speckle distributions are both well described by a gamma function. Similarly, Weatherbee *et al*.^24^, compare a Rayleigh distribution to a K-distribution to describe the OCT amplitude distribution. Their results show that for a large number of scattering particles both Rayleigh and K-distributions correspond well with the experimental speckle distribution, while for a small number of scattering particles per voxel (N = 2.4 and N = 0.6) a K-distribution appears more appropriate. The samples used in our study far exceed such particle densities so that statistics corresponding to the Rayleigh distribution are expected. Hillman *et al*. have previously shown that for samples with N > 10 randomly distributed scattering particles, the contrast of the OCT amplitude distribution is equal to 0.52, consistent with a Rayleigh distribution. Our experimental results confirm these findings and we conclude that the OCT amplitude distribution for homogenous samples is well described by a Rayleigh distribution, and that consequently the amplitude variance is proportional to the mean amplitude. While we found the amplitude distribution for homogeneous samples to be Rayleigh distributed, the amplitude distribution of inhomogeneous samples may differ, conceivably providing a measure for tissue heterogeneity. These findings yield the first rigorous analytical framework describing the OCT amplitude distribution and its variance. For practical use of OCT amplitude distribution statistics as a quantitative measure for structural properties, we have studied the influence of the axial size and position of the ROI window on the obtained amplitude distribution. We found that when sampling the amplitude distribution from a ROI window of fixed size which is moved in the axial direction, the amplitude distribution continues to have a Rayleigh distribution. Conversely, sampling the amplitude distribution from a ROI window which is successively increased in depth, the amplitude distribution increasingly deviates from a Rayleigh distribution the larger the window becomes: by incorporating areas of strongly attenuated OCT signals, the tail of the distribution becomes more pronounced.

To further validate our description of the optical properties *µ*_*s*_ and *µ*_*b,NA*_, we compared calculated with OCT-derived optical properties for controlled sample of silica microspheres by obtaining the attenuation coefficient and amplitude variance from OCT data and carefully calibrating for system parameters. For the scaling between amplitude variance and *µ*_*b,NA*_ calibration factor α(z) was obtained from sample G and used for all samples. The experimentally determined values are in good agreement with the calculated curves. Here, we assumed minimal signal contribution by multiply scattered light in the reported samples, so that *µ*_*OCT*_ ≈ *µ*_*s*_. This assumption follows from previous work where we show that the amount of multiple scattering contributing to the signal only has a significant influence (>10%) in samples with a high *µ*_*s*_ and high scattering anisotropy (g), which is only the case for sample H. Finally, the calculated and experimental results indicate that DRM optical properties, for samples with approximately >10 scattering particles per voxel, (*µ*_*s*_ and *µ*_*b,NA*_) are sensitive to sub-resolution changes in size and concentration of the scattering particles: for homogenous samples the backscattering coefficient is proportional to the variance of the amplitude distribution. This relation could be used as an additional parameter to differentiate between samples with equal *µ*_*s*_. Moreover, the results suggest that *µ*_*s*_ and *µ*_*b,NA*_ could be used to obtain sub-resolution size information of the scattering particles^25^. However, the calculated curves show that this approach is hampered by the periodic dependence of *µ*_*b,NA*_ as a function of optical size. For determination of sizes that range over multiple periods (Fig. 4) the results would not converge to a single solution. This problem may be overcome using measurements at a large range of wavelengths^26^.

The presented derivation of the OCT signal applies to homogeneous samples of mono-disperse DRM, which is not directly applicable to tissue. Likely, the scattering properties of tissue are more realistically described using a Continuous Random Medium formalism^27,28^. However, the parameters for CRM modeling (refractive index correlation length, refractive index variance, ‘fractal parameter’) are not easy to control in samples made for validation. Our first step towards the extraction of quantitative measures of tissue organization from OCT measurements is modelling and validating samples of DRM. The scattering coefficients can reliably be described with the Percus-Yevick solution for the pair correlation function (organization) and Mie theory (scattering). The input parameters for these calculations are well known and by comparing theoretical predictions with. experimental results we are able to validate our approach in this paper as well as in our previous work^7^. The next step, towards a model for tissue scattering, is to find expressions for the scattering properties of poly-disperse samples. For bi-disperse samples, analytical solutions may still be available allowing thorough validation. Samples with a broad distribution of spherical particles, such as Intralipid samples, form an interesting case since it may be possible to describe them both by formalisms based on (poly-disperse) DRM and based on CRM. Our expectation is that ultimately, this will allow better physical interpretation of CRM-derived properties. Although limited to DRM, the presented derivation of the OCT signal yields novel physical insight on the relation between structural properties of a sample on the OCT-derived optical properties. These results may have implications for clinical research, as the ability to probe size and organization of cells and cellular structures is essential to assessing the function and health of biological tissues^9,29^.

## Methods

### Sample preparation

Mono-disperse silica microspheres (Kisker Biotech, Steinfurt, Germany) with mean diameters of 0.47, 0.70, 0.91, 1.60 µm (determined with transmission electron microscopy (Philips CM-10)^23^) were suspended in water to prepare samples with scattering coefficients of 4.0 mm^−1^ (±0.5 mm^−1^) and 6.0 mm^−1^ (±0.5 mm^−1^). The required silica microsphere volume fractions were calculated using inverse Mie theory (differential scattering cross section) and the Percus-Yevick structure factor equation (10), given the density of the silica microspheres by the manufacturer (2.0 gram/cm^3^). Sodium dodecyl sulfate (0.03 mM) was added to the suspensions to prevent aggregation of the silica microspheres^30^. An overview of the prepared samples and the average number of microspheres within the OCT coherence volume (equation (1)) is given in Table 1.

### OCT experiments and data analysis

OCT data were recorded using a Santec IVS 2000 swept-source OCT system, with a center wavelength of *λ*_*0*_ = 1309 nm, ~100 nm sweep range, with a sweep rate of 50 kHz. The FWHM measured axial resolution was 18 μm in air. The *NA*, FWHM beam waist (ω_0_) and Rayleigh length of the system were determined based on a knife-edge measurement and were 0.02, 25 μm and 960 μm, respectively. The focus position was aligned with the zero delay position. The samples were measured in a 1 mm quartz cuvette, with the inner boundary of the cuvette placed at 1 mm (+/−50 µm) from zero delay. All samples were measured without moving the cuvette, to ensure no change in optical geometry in between measurements. From every sample 10 cross section images (B-scan) were recorded. Every B-scan contained 1000 adjacent A-lines over a lateral scanning range of 5 mm.

The attenuation coefficient of the OCT signal in depth was obtained by Non-Linear Least Squares fit (custom written code in LABVIEW 2013, National Instruments) of a single exponential decay in which the amplitude *A* and the decay constant *µ*_*fit*_ were free running parameters. The average noise level, directly at the backside of the cuvette, was added to the fit as a fixed offset. The region of interest (ROI) over which the fit was performed was chosen manually. The final *µ*_*fit*_ value was obtained as the mean value of 100 automatically performed fits, in which the ROI boundaries were varied within 5%. The obtained attenuation coefficient of the samples was corrected for the group refractive index of water (*n* = 1.34) and system parameters (point spread function and sensitivity roll-off) as described in Ref ^7^. This procedure was carried out for five B-scans per sample from which the mean value and standard deviation of *µ*_*fit*_ were obtained.

Speckle variance, contrast and mean of all samples were calculated from the linear OCT data using a custom written JAVA code. The ROI window was set at ~0.03 mm optical path length below the cuvette wall-sample interface and was ~ 0.08 mm (optical path length) in depth (Supplementary Information Section IX), except for the experiments presented in Fig. 7 in which the window is enlarged in depth with fixed upper boundary. To avoid effects of attenuation within the ROI, the ROI size in depth is smaller than 1 mfp (~ 0.15 mm optical path length) unless specified otherwise. The lateral size of the ROI window was chosen such to ensure a speckle contrast around 0.52 corresponding to fully developed speckle^13,14^. The mean and standard deviation of the speckle variance within the ROI window was obtained from ten B-scans per sample.

The obtained speckle variance values from the experimental data were scaled to the calculated *μ*_*b,NA*_ curve using a scaling factor calculated using sample G. This 0.91 μm diameter silica microsphere sample was chosen for calibration because of the low standard deviation of the measured speckle variance.

### Theoretical calculations

Theoretical *µ*_*s*_ and *µ*_*b,NA*_ curves were calculated using a custom written Labview code (Labview 2013, National Instruments). Mie calculations from ref ^31^. were implemented to calculate the differential scattering cross section for each particle from the mean diameter of the particles determined with transmission electron microscopy (Table 1), integrated over spectral bandwidth. Both the refractive index of the medium and silica microspheres varied with wavelength, with values of 1.324 for water^32^ and 1.425 for the silica microspheres at 1300 nm^7,33^. The concentration-dependent *µ*_*s*_ (equation (10)) and *µ*_*b,NA*_ (equation (9)) were calculated by combining Mie-calculated *σ*_*S*_ *(θ)* with the structure factor S(θ). Mean particle diameter, refractive indices, wavelength, volume fraction, and system NA were used as input parameters.

### Data availability

The datasets generated during and/or analysed during the current study are available from the corresponding author on reasonable request.

## Electronic supplementary material

Supplementary Information
