# Supplementary material for: OCT Amplitude and Speckle Statistics of Discrete Random Media
Source: Sci Rep. 2017 Nov 1;7:14873. doi: 10.1038/s41598-017-14115-3 (PMC5665955; doi:10.1038/s41598-017-14115-3)
Supplement: Supplementary file 1 — Supplementary Information [file 41598_2017_14115_MOESM1_ESM.pdf]

OCT Amplitude and Speckle Statistics of Discrete Random Media

Mitra Almasian,[a] Ton G. van Leeuwen,[a] Dirk J. Faber[a*]

d.j.faber@amc.uva.nl

Academic Medical Center, University of Amsterdam, Department of Biomedical Engineering and Physics, Amsterdam, The Netherlands

SUPPLEMENTARY INFORMATION

I – OCT geometry and signal

Consider a medium containing hard spheres of a single size, where the position of the i'th particle is given by $\delta(\boldsymbol{r} - \boldsymbol{r_i})$; measured from a given reference particle (see Figure SI-1 below). These spheres can touch, but not overlap in space. This medium is imaged using an OCT system with the reference arm length ('zero delay' matched to the position of a reference particle located at depth z_b below the boundary. The scattered light is collected at the lens in the detection arm, with *R* the distance between reference particle and lens.

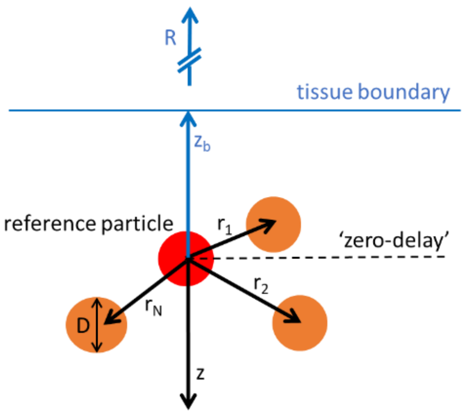

Figure SI-1: OCT geometry. The optical path length in the reference arm is matched to the position of a reference particle in the sample ('zero delay'). The sample consists of identical randomly placed spheres with position $\boldsymbol{r}_i$ *with respect to the reference particle. The distance R is between the reference particle and the detection lens in the sample arm.*

The (complex) scattered field from a volume containing N particles is given by $E_s = \sum_{i=1}^{N} E_i$. For identical particles (equal size and refractive index), the scattering efficiency of the individual particles is the equal, but the amplitudes and phases of the scattered fields depend on the individual particle positions. The total field at the detector is given by $E_D = E_R + \sum_{i=1}^{N} E_i$ were E_R is the field scattered from the reference mirror (phase-matched to the reference particle).

The real part of the (Time-Domain) OCT signal as function of depth *x(z)* is obtained from the detector current

$$i_{DET}(z) \propto \langle |E_D|^2 \rangle = I_S(z) + I_R(z) + 2\langle E_R \sum_{i=1}^{N} E_i \rangle \qquad (1a)$$

$$x(z) \propto \langle E_R \sum_{i=1}^{N} E_i \rangle \qquad (1b)$$

where the brackets denote averaging over the detector response time. I_S and I_R are intensities from sample and refernce arm, respectively. Removing these DC-terms yields x(z) as a cosine-modulated signal with zero-mean and non-zero variance that encodes the sample reflectivity. The imaginary part *y(z)* is sine-modulated at the same frequency. In OCT, conventionally the amplitude $A(z) = \sqrt{x^2(z) + y^2(z)}$ is plotted in a logarithmic grayscale image. Both mean and variance of A(z) are non-zero.

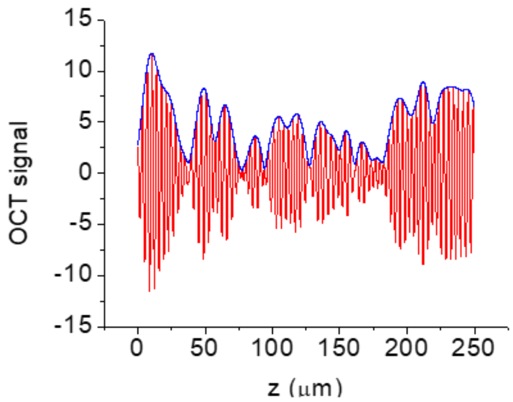

Figure SI-2: simulated OCT signal as function of depth of a sample containing many randomly placed reflectors. The red curve shows the real part x(z) *of the complex OCT signal, cosine modulated with zero mean and non-zero variance. The imaginary part* y(z) *is not shown. The blue curve shows the amplitude A(z).*

Figure SI-2 shows simulations of $x(z)$, red curve, and $A(z)$, blue curve, from a sample containing many identical, randomly placed reflectors (N>10, see SI-IV). The number of reflectors is too high to resolve their individual positions: the information about particle density and scattering strength is encoded in the variance of $x(z)$, and mean and variance of $A(z)$.

II – Time Domain vs. Spectral domain OCT

The analysis in SI-I assumed detection of the OCT signal in the spatial domain (e.g. “Time-domain OCT”) rather than in the spatial frequency domain (Spectral domain OCT). The analysis however, remains the same because the time-domain and spectral domain signal are reversibly connected through a Fourier transformation. To illustrate, Figure SI-3A shows a simulated ‘raw spectrum’ corresponding to a single reflector, i.e. a cosine modulated in source spectrum with the position of the reflector encoded in the modulation frequency (a real signal). The result of the Fourier transform of this raw spectrum is complex. Panel B shows the result if the real part of the complex FT is calculated the signal corresponding to the red curve is obtained; if the amplitude of the complex FT is calculated the envelope (blue curve) is obtained. Note that in practice, the square of the amplitude is often calculated directly from the Power Spectrum of the raw spectrum instead of via FT; and log(A^2) is converted to a grayscale image.

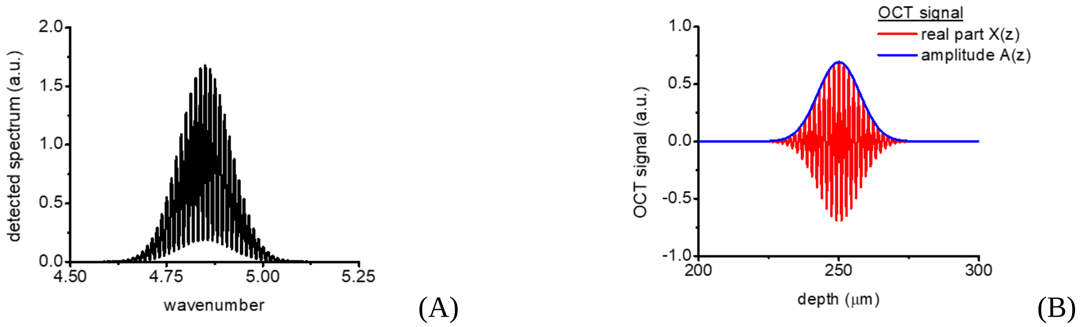

Figure SI-3: (A) Simulated raw spectrum in Spectral Domain OCT corresponding to a single reflector. (B) The Fourier transform of this spectrum yields a complex signal with real part x(z), red curve and amplitude A(z), blue curve.

III – OCT Speckle

The (complex) scattered field from a volume containing N particles is written as $E_s = \sum_{i=1}^{N} E_i$. Both the amplitude and phase of E_i can be considered as random variables with no dependency on each other. Therefore, the statistics of the scattered field and according to Supplementary Eq. 1B, $x(z)$ follow that of a random phasor sum as is commonly found in speckle phenomena.[1] Indeed, the random amplitudes and phases of the different scattering elements within a detection volume give rise to the static speckle pattern found in OCT images. For a large number of scatterers $x(z)$ and $y(z)$ follow a normal distribution (by the Central Limit theorem) with zero mean and non-zero variance. The amplitude $A(z) = \sqrt{x^2(z) + y^2(z)}$ is Rayleigh distributed (Supplementary Eq. 2A) with mean $\langle A \rangle$ and variance σ_A^2 determined by the variance of the underlying real and imaginary components $\sigma_x^2 = \sigma_y^2 = \sigma_{x,y}^2$ (Supplementary Eq. 2B):

$$p(A) = \frac{A}{\sigma^2} e^{-A^2/\sigma_A^2} \tag{2A}$$

$$\langle A \rangle = \sqrt{\frac{\pi}{2} \sigma_{x,y}^2}; \qquad \sigma_A^2 = \left(2 - \frac{\pi}{2}\right) \sigma_{x,y}^2 \tag{2B}$$

This also yields the familiar result that contrast in OCT, when defined as the ratio of the standard deviation over the amplitude of the OCT signal is $\sqrt{4/\pi - 1} \approx 0.52$.[2,3]

Note that when the OCT envelope is calculated as the power spectrum of a signal acquired in the spatial frequency domain, the distribution of this signal p(P) follows an exponential distribution, and the contrast is unity (see SI-II).

IV – the real part of the OCT signal x(z)

The information about particle density and particle scattering strength is encoded in the variance of $x(z)$, see SI-I, and via Supplementary Eq. 2B also in the mean and variance of the OCT amplitude $A(z)$. In order to express $\langle A\rangle$ and σ_A^2 in terms of sample scattering properties, we first derive an expression for $x(z)$ that can be used to calculate σ_x^2 around the position of the reference particle:

$$\sigma_x^2 \equiv \langle x^2(z)\rangle - \langle x(z)\rangle^2 = \langle x^2(z)\rangle \quad (3)$$

The last equality holds because for discrete random media, the mean of $x(z)$ is zero. We assume N identical particles in the probe volume, where the scattered field in the far field of the n'th particle can be written as[4]:

$$E_{s,n} = E_{in}\frac{f(\theta,\varphi)}{kR}e^{i\phi_n} \quad (4)$$

Where E_{in} is the input field which we assume identical for each particle under the 1st Born approximation; $f(\theta,\varphi)$ is the *scattering amplitude* of the particles (in general a complex number), ϕ_n is the phase of the scattered field which depends on the position of the particle, k is the wavenumber $k=2\pi/\lambda$ where λ is the wavelength; and R is the distance to point of evaluation. Since the distances between the particles is much smaller than the distance from the reference particle to the lens (See SI-I and Figure SI-1) we take identical R for all particles.

In the following, we express the phase of the scattered field with respect to the reference particle. The phase difference between fields scattered from 2 arbitrary particles can be written as $\Delta\phi = \mathbf{q}\cdot\mathbf{r}$ where $\mathbf{q}$ is the wavevector $\mathbf{k}_{out} - \mathbf{k}_{in}$, $|\mathbf{q}| = 2k\sin\frac{1}{2}\theta$ with θ the scattering angle (angle of observation); and $\mathbf{r}$ is the vector connecting both particles. Consequently, for the n'th particle we write $\phi_n = \mathbf{q}\cdot\mathbf{r_n}$ and the computed value of x is assigned to z_b (the position of the reference particle).

Since the reference particle is matched to the reference arm, $\mathbf{q}\cdot\mathbf{r_n}$ also equals the phase difference between the scattered field and the reference arm field. This allows us to express the relative contribution of the field scattered from n'th particle trough the complex coherence function $\gamma(\mathbf{q}\cdot\mathbf{r_n})$. For an example of the real part of the complex coherence function see Figure SI-3B.

Finally, the scattered field contribution needs to be evaluated over the solid angle corresponding to the detection numerical aperture Ω_{NA}(i.e. of the lens in the sample arm). Combining terms, we expand Supplementary Eq. 1b as:

$$x(z_b) \propto Re\left\{\int_{\Omega NA}\sum_i \gamma(\vec{q}\cdot\vec{r_i})\, e^{i\vec{q}\cdot\vec{r}_i}\frac{f(\theta,\varphi)}{kR}d\Omega\right\} \quad (5)$$

Only particles within the coherence volume V_c contribute to the signal. The axial dimension of this cylindrical volume is in the order of the coherence length, the lateral radius in the order of the probe beam waist when evaluated at the focus position. Sugita et al [Ref 15 of the manuscript] derived the following expression to which we adhere:

$$V_C = \frac{4\pi{\omega_0}^2\sqrt{2\ln(2)}L_C}{6} \quad (6)$$

Where L_C is the coherence length and ω_0 the 1/e intensity waist of the illuminating beam. We use this to make a further simplification of Supplementary Eq. 5 by assuming that only particles within V_C contribute with equal weight to the OCT signal. Therefore we can omit the complex coherence function if we sum only over these N particles:

$$x(z_b) \propto Re\left\{\int_{\Omega NA} \sum_{i=1}^{N} e^{i\vec{q}\cdot\vec{r}_i} \frac{f(\theta,\varphi)}{kR} d\Omega\right\} \quad (7)$$

where N = ρV_C and ρ is the average particle density (constant for the homogeneous medium assumed here).

V – mean squared real OCT signal $\langle x^2(z)\rangle$

Starting with Supplementary Eq. 7 for the real part of the OCT signal we write the mean square of the real OCT signal as:

$$\langle x(z_b)^2\rangle \propto Re\left\{\int_{\Omega NA} \langle \sum_{i=1}^{N}\sum_{j=1}^{N} \frac{f^2(\theta,\varphi)}{k^2R^2} e^{i\vec{q}\cdot\vec{r}_i} e^{-i\vec{q}\cdot\vec{r}_j}\rangle \, d\Omega\right\} \quad (8)$$

This equation can be further simplified by using the definition of the differential scattering cross section of the (identical) particles, $\sigma_{scat}(\theta,\varphi) = f(\theta,\varphi)^2/k^2$ [Ref 22 of the manuscript] which can be taken out of the ensemble average. Further, the integration over solid angle can be written in spherical coordinates, yielding:

$$\langle x(z_b)^2\rangle \propto Re\left\{\int_0^{2\pi}\int_{(\pi-NA)}^{\pi} \sigma_{scat}(\theta,\varphi)\langle \sum_{i=1}^{N}\sum_{j=1}^{N} e^{i\vec{q}\cdot\vec{r}_i} e^{-i\vec{q}\cdot\vec{r}_j}\rangle \, sin\theta d\theta d\varphi\right\} \quad (9)$$

For spherical particles as considered here, the differential scattering cross section does not depend on the azimuthal angle φ, and the integral over φ simply yields a factor 2π.

The double sum within chevrons ⟨...⟩ accounts for all phase differences between the particles contributing to the signal and is known from statistical physics as the structure factor.[5] More precisely:

$$S(q) = \frac{1}{N}\langle \sum_{i=1}^{N}\sum_{j=1}^{N} e^{i\vec{q}\cdot\vec{r}_i} e^{-i\vec{q}\cdot\vec{r}_j}\rangle \quad (10)$$

Using $N = \rho V_C$ from SI-IV where V_C is the coherence volume and ρ is the average particle density and q is the scattering vector with magnitude 2ksin½θ (see SI-IV). Consequently, the structure factor may be written as function of θ for convenience. Thus, and since both the differential cross section and structure factor are real numbers:

$$\langle x(z_b)^2\rangle = \sigma_x^2(z_b) \propto \rho V_C \times 2\pi \int_{(\pi-NA)}^{\pi} \sigma_{scat}(\theta)S(\theta) \, sin\theta d\theta \quad (11)$$

The equality holds based on Supplementary Eq. 3.

The structure factor quantifies the effect of organization of scatters in the sample on the scattering pattern (hence its name). For discrete random media, it is closely related to the pair-correlation $g(\Delta r)$ function through a Fourier transform relationship:

$$S(q) = 1 + \rho \int g(\Delta r) e^{i\vec{q}\cdot\Delta\vec{r})} d\Delta\vec{r} \quad (12)$$

Where the pair-correlation function is interpreted the distribution of particle separations Δr. Note that both the pair-correlation function and structure factor are functions of particle density ρ. Supplementary Eq. 11 demonstrates that the variance of the real OCT signal σ_x^2 and therefore by Supplementary Eq. 2B also the mean $\langle A\rangle$ and variance σ_A^2 of the OCT amplitude signal encode the scattering strength of the particles (differential cross section term) and the particle density and organization (structure factor term).

VI – interpretation as optical coefficient

To facilitate interpretation of Supplementary Eq. 11, we first note the definitions[4] of the (total) scattering cross section (units [m^2]) of a single spherical particle (Supplementary Eq. 13) and scattering coefficient (units [m^{-1}]) of a medium containing such particles (Supplementary Eq. 14):

$$\sigma_{scat} = 2\pi \int_0^{\pi} \sigma_{scat}(\theta)\, sin\theta d\theta \tag{13}$$

$$\mu_s = \rho\sigma_{scat} \tag{14}$$

Comparing Supplementary Eqs 11 and 13, we find that the integral is weighted with the dimensionless structure factor to account for organization in the sample. Moreover, integral boundaries are limited to the detection NA, which leads to the following cross section and coefficient 'in the backscatter direction, within the detection NA':

$$\sigma_{b,NA} = 2\pi \int_{(\pi-NA)}^{\pi} \sigma_{scat}(\theta)S(\theta)\, sin\theta d\theta \tag{15}$$

$$\mu_{b,NA} = \rho\sigma_{b,NA} \tag{16}$$

Thus, at a given location in the sample, the variance of the real part of the OCT signal σ_x^2 and the variance of the envelope signal are proportional to $\mu_{b,NA}$; the mean amplitude $\langle A\rangle$ is proportional to $\sqrt{\mu_{b,NA}}$.

VI – OCT amplitude vs. depth.

The numerical aperture in Supplementary Eq. 15 could theoretically be expanded to collect *all* scattered light. In that case, the expressions for the scattering cross section and scattering coefficient of the discrete random medium become:

$$\sigma_{scat,medium} = 2\pi \int_0^{\pi} \sigma_{scat,particle}(\theta)\, S(\theta) sin\theta d\theta \tag{17}$$

$$\mu_{s,medium} = \rho 2\pi \int_0^{\pi} \sigma_{scat,particle}(\theta)\, S(\theta) sin\theta d\theta \tag{18}$$

With subscripts 'medium' and 'particle' added for emphasis but omitted henceforth.

In SI-I to SI-V it was assumed that the reference arm is matched to a reference particle at arbitrary depth z_b, and that under the 1st Born approximation, all particles within the coherence volume V_C around z_b experience the same input field. The amplitude of this illuminating field however decreases in amplitude with increasing z_b because of losses due to light scattering and absorption (the latter is neglected in the analysis in this SI). Likewise, part of the light scattered from V_C is scattered on the way back to the sample boundary; it is assumed that this light escapes the detection NA and does not contribute. In other words, only single scattered light is considered, for which the attenuation of intensity can be described by the Lambert-Beer law, with the μ_s of Supplementary Eq. 18 as exponential decay constant. Additional depth-dependent weighting terms exist, such as the confocal point spread function (the illuminating field will be weaker if the reference particle is chosen outside the focal region) and, specifically for Spectral Domain OCT, the sensitivity-rolloff in depth. For a thorough discussion of these factors we refer to our earlier work [[6]] and references therein.

In a *Time-Domain system,* the moving reference arm would vary the probe depth z_b and the coherence volume around it to build up an A-line. In this case, the subscript 'b' may be dropped and the OCT A-line, defined as $\langle A(z)\rangle$ and variance $\sigma_A^2(z)$ are written as:

$$\langle A(z)\rangle_{TD} = \sqrt{\alpha_{TD}(z)\frac{\pi}{2}\mu_{b,NA}V_C\, exp(-2\mu_s z)} \tag{19}$$

$$\sigma_A^2(z)_{TD} = \alpha_{TD}(z)\left(2-\frac{\pi}{2}\right)\mu_{b,NA}V_C\, exp(-2\mu_s z) \tag{20}$$

Were depth z is measured from the sample boundary. Here μ_s is given by Supplementary Eq. 18, and μ_{bNA} by the analysis SI-IV. The factor 2 in accounts for scattering losses to and from the coherence volume. The term $\alpha_{TD}(z)$ accounts for scaling factors such as power-to-current efficiency of the detector, but also depth dependent losses, most notably the confocal point spread function – either using a static focus or dynamic focusing. Importantly, $\alpha_{TD}(z)$ only contains parameters related to the OCT system (not the sample) and can thus in principle be calibrated to allow for absolute measurements of μ_{bNA}.

In a *Spectral-Domain system* the zero-delay position is usually not located within the sample but at some position outside. This does not change our analysis since it will only lead to a fixed phase difference between zero delay and the reference particle. Particles will contribute to the signal as long as the distance between zero-delay and the reference particle is within the instantaneous coherence length of the system, which is determined by the spectral resolution. We therefore only slightly modify Supplementary Eqs. 19 and 20:

$$\langle A(z)\rangle_{SD} = \sqrt{\alpha_{SD}(z)\frac{\pi}{2}\mu_{b,NA}V_C\, exp\big(-2\mu_s(z-z_0)\big)} \tag{21}$$

$$\sigma_A^2(z)_{SD} = \alpha_{SD}(z)\left(2 - \frac{\pi}{2}\right)\mu_{b,NA}V_C\, exp\big(-2\mu_s(z - z_0)\big) \quad (22)$$

Were depth z is measured from zero-delay; and z0 is the distance between zero-delay and the sample boundary. The term $\alpha_{SD}(z)$ accounts for scaling factors such as power-to-current efficiency of the detector, but also depth dependent losses, most notably the confocal point spread function and sensitivity roll-off with depth inherent to SD-OCT systems. Again, $\alpha_{SD}(z)$ only contains parameters related to the OCT system (not the sample) and can thus in principle be calibrated to allow for absolute measurements of μ_{bNA}.

VII – scaling of optical properties with volume fraction

Figure SI-4 shows the volume fraction-dependent (Eq. 9 and Eq. 10) and volume fraction-independent calculations for $\mu_{B,NA}$,μ_s, $\mu_{B,NA}/\mu_s$, *anisotropy* as a function of optical size (D·k). For the latter calculations the structure factor is set to unity. Thus, the f_v-independent $\mu_{B,NA}/\mu_s$ and anisotropy curves do not change with volume fraction. For the f_v -dependent calculation all shown plots change with volume fraction since the structure factor, which serves as a weighting factor on the angular scattering pattern (phase function) is a function of f_v. (see also SI-VIII).

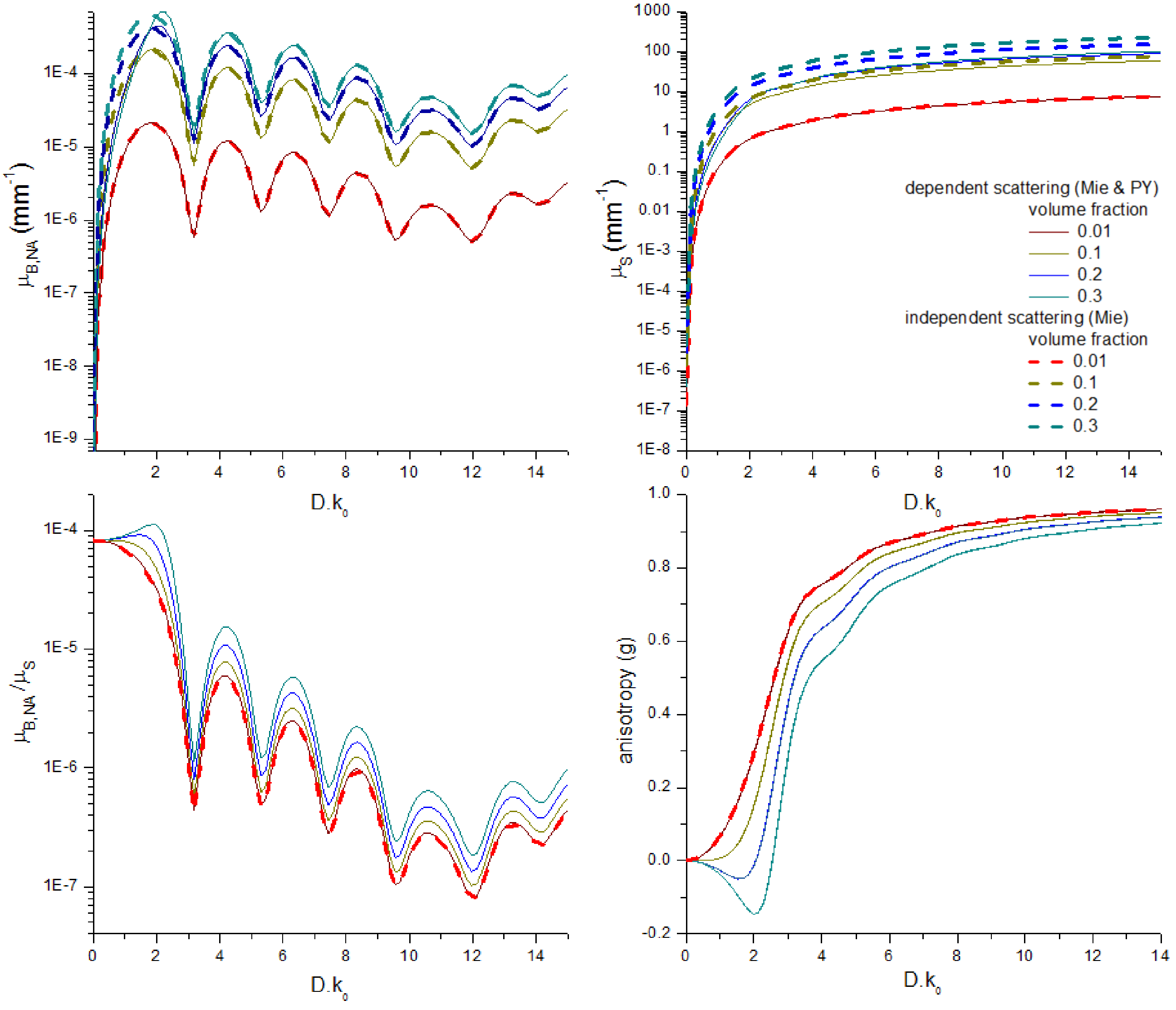

Figure SI-4: Calculated backscattering coefficient ($\mu_{B,NA}$) (and scattering coefficient (μ_s), $\mu_{B,NA}/\mu_s$ and anisotropy as a function of optical particle diameter (D. k_0, $k_0=2\pi/\lambda_{0,vaccuum}$) for volume fractions of 0.01, 0.1, 0.2 and 0.3 for a center wavelength λ_0 = 1300 and bandwidth of $\Delta\lambda$ = 100 nm. The solid lines are depict the concentration-dependent calculation using Eq.11 and Eq. 12. The dotted lines show the concentration-independent calculations, these are the MIE solutions. For these calculations the structure factor $S(\theta)$ in Eq. 11 and 12 is set to unity.

VIII – phase function change with concentration/volume fraction

Our analysis accounts for the phase differences between the fields scattered by the individual particles in the sample. This analysis gives rise to a *structure factor* (see SI-V and Supplementary Eq. 10) that directly influences the angular light scattering (compare Supplementary Eqs. 13 and 15).

Applied to the low-NA backscattering geometry as described in our experiments (NA=0.02; θ_{NA} ~ 1°), for increasing volume fraction the NA-integrated part of the backscattered intensity increases approximately linearly with volume fraction, whereas the total scattered fraction decreases with volume fraction [[6]].

The effect of the structure factor on angular scattering is illustrated below (*$D=0.91\ \mu m$; $\lambda_0 = 1300\ nm$; $n_{part} = 1.425$; $n_{med} = 1.324$*). We calculate the phase function, which by definition is normalized on the full solid angle. Blue curves show the phase function without inclusion of a structure factor calculated as:

$$p_{Mie}(\theta) = \frac{\sigma_s(\theta)}{2\pi \int_0^{\pi} \sigma_s(\theta) sin\theta d\theta} \tag{23}$$

Where $\sigma_s(\theta)$ is the differential scattering cross section for a single particle obtained with Mie theory. Red curves show the phase function when the structure factor is included:

$$p_{Mie-PY}(\theta) = \frac{\sigma_s(\theta)S(\theta)}{2\pi \int_0^{\pi} \sigma_s(\theta)(\theta) sin\theta d\theta} \tag{24}$$

Where $S(\theta)$ is the Percus-Yevick structure factor appropriate for Discrete Random Media.[5,7] All blue curves are identical. For the red curves (including the structure factor), with increasing volume fraction the amplitude of the phase function in the forward direction decreases, the amplitude in the backward direction increases, and the overall shape of the phase function becomes broader. This corresponds to a decrease of scattering anisotropy g (the average cosine of the scattering angle) as is also observed in SI-VII, Figure SI-4.

f_v = 0.01

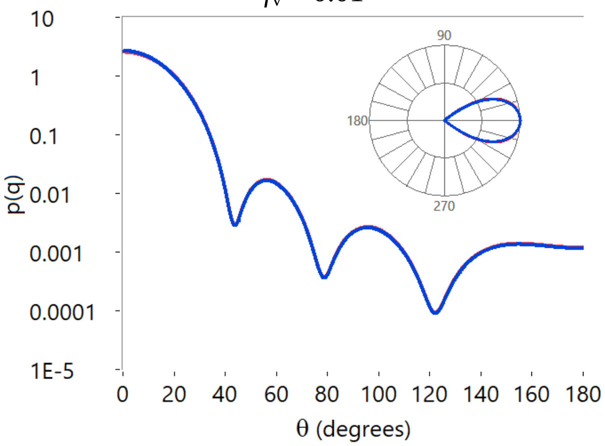

f_v = 0.1

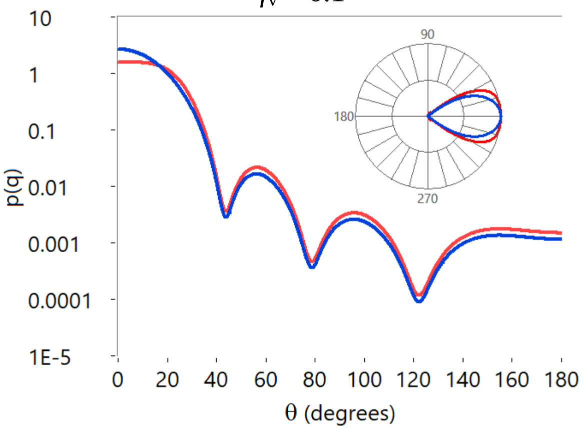

f_v = 0.2

f_v = 0.3

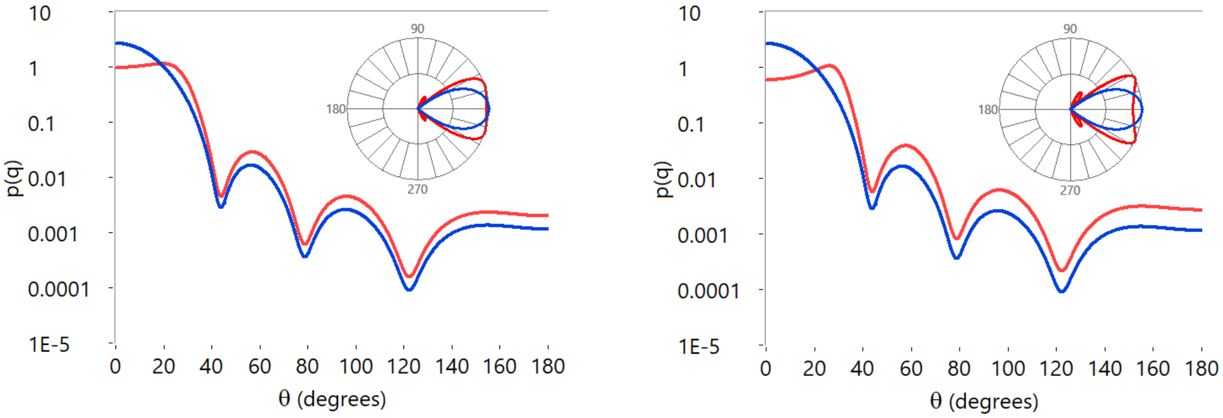

Figure SI-5: The phase function [4] (scattered intensity normalized on solid angle) is calculated for volume fractions of 0.01, 0.1, 0.2 and 0.3. using Mie theory (D=0.91 µm; λ_0= 1300 nm; n_{part} = 1.425; n_{med} = 1.324) without (blue curves) and including the structure factor (red curves) as calculated from the Percus Yevick approximation. Note that our experiments did not exceed volume fractions of 0.06.

IX – region of interest selection for speckle distribution analysis

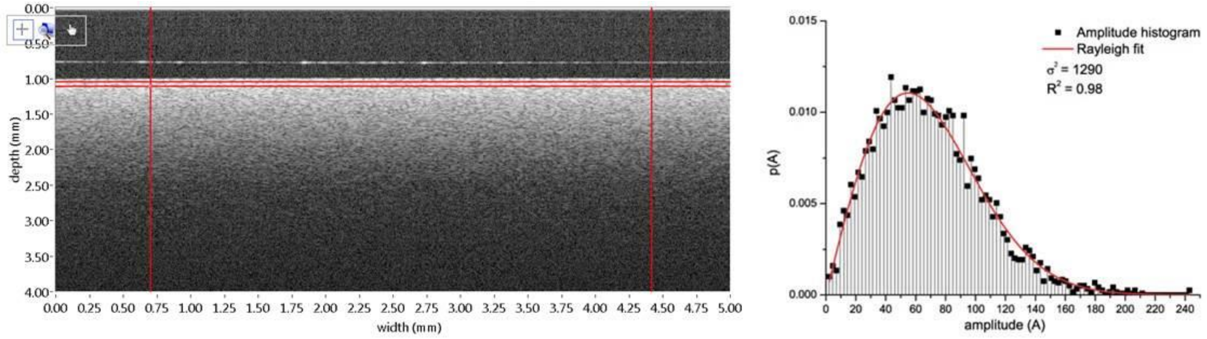

Figure SI 6: Region of interest selection for amplitude distribution analysis. Left panel shows an OCT B-scan of a sample. The area between the four red lines is the region of interest from which the distribution of the amplitude, and the mean, variance and contrast are obtained. The right panel shows the histogram of the amplitude distribution from the region of interest plotted in the left panel. The amplitude distribution is fitted with a Rayleigh distribution (Eq. 3 of the manuscript), for which the values for the R^2 and variance (σ^2) are given.

References

1. J. W. Goodman, *Speckle Phenomena in Optics: Theory and Applications*, Roberts and Company Publishers, Greenwood Village (2007).
2. T. R. Hillman et al., "Correlation of static speckle with sample properties in optical coherence tomography," *Opt. Lett.* **31**(2), 190 (2006).
3. B. Karamata et al., "Speckle statistics in optical coherence tomography SPECKLES IN OPTICAL COHERENCE," 593–596 (2005).
4. H. C. van der Hulst, *Light scattering by small particles*, Dover publications, New York (1957).
5. J.-P. Hansen and I. R. McDonald, *Theory of simple liquids*, Elsevier (1990).
6. M. Almasian et al., "Validation of quantitative attenuation and backscattering coefficient measurements by optical coherence tomography in the concentration- dependent and multiple scattering regime," *J. Biomed. Opt.* **20**(12) (2015).
7. J. K. Percus and G. J. Yevick, "Analysis of Classical Statistical Mechanics by Means of Collective Coordinates," *Phys. Rev.* **110**(1), 1–13 (1958).
